# Extracorporeal membrane oxygenator (ECMO) and ventricular assist device (VAD) activity of a tertiary cardiothoracic centre: survival rates and length of ITU stay

**DOI:** 10.1186/cc14360

**Published:** 2015-03-16

**Authors:** D Sarridou, CP Walker, N Rashid, D Heaton, I McGovern, N Marczin, J Mitchell

**Affiliations:** 1 The Royal Brompton & Harefield NHS Foundation Trust, London, UK; 2 Imperial College, London, UK

## Introduction

Harefield Hospital accepts patients as a tertiary centre for end-stage heart and respiratory failure for the south of England. Interventions include VA-ECMO, VV-ECMO as a bridge to lung transplantation, left ventricular assist devices (LVAD) as a bridge for heart transplantation, right ventricular assist devices (RVAD) and BIVADs. The aim of this review was to identify the total number of such patients, analyse the individual length of ITU stay and calculate survival and mortality rates for each intervention.

## Methods

Patients consisted of six groups: Group 1: VA ECMO, Group 2: VV-ECMO, Group 3: LVAD, Group 4: RVAD, Group 5: BIVAD, Group 6: combination of devices. Data were extracted from the Perfusion Department records and the intensive care dataset from 2011 to 2013. Data included length of ITU stay, outcome, indication for device insertion and device-related major complications.

## Results

Forty patients were identified. Twenty-nine were male and 11 female. Group 1 included 22 patients, Group 2: two patients, Group 3: four patients, Group 4: four patients, Group 5: zero, Group 6: eight patients treated with various combinations of ECMO or ventricular assist devices. ITU stay varied from 1 day to a maximum of 6 months intermittently for one patient. Duration of ITU stay for all 40 patients was 1,052 days with an average of 26.3 days per patient. Sixteen patients survived and were discharged to the Transplant Unit. Twenty-four patients died, putting the survival rate at 40% for this group.

## Conclusion

This review demonstrates that the majority of these patients occupied intensive care beds for a prolonged period of time and despite the use of advanced support devices survival rates were significantly lower than mortality rates.

